# Assessment of meniscal extrusion with ultrasonography: a systematic review and meta-analysis

**DOI:** 10.1186/s43019-024-00236-3

**Published:** 2024-10-28

**Authors:** Khalis Boksh, Duncan E. T. Shepherd, Daniel M. Espino, Jenna Shepherd, Arijit Ghosh, Randeep Aujla, Tarek Boutefnouchet

**Affiliations:** 1 Department of Biomedical Engineering, University of Birmingham, Birmingham, UK; 2 Leicester Academic Knee Unit, University Hospitals of Leicester NHS Trust, Leicester, UK; 3 Department of Trauma & Orthopaedics, University Hospitals of Birmingham NHS Trust, Birmingham, UK

**Keywords:** Meniscal extrusion, Ultrasonography, Osteoarthritis, Meniscus pathology, Correlation

## Abstract

**Background:**

Magnetic resonance imaging (MRI) is the imaging of choice for meniscal extrusion (ME). However, they may underappreciate the load-dependent changes of the meniscus. There is growing evidence that weight-bearing ultrasound (WB US) is more suitable, particularly in revealing occult extrusion. We therefore perform a systematic review and meta-analysis on the validity and reliability of US in diagnosing extrusion. Furthermore, we explored whether it detects differences in extrusion between loaded and unloaded positions and those with pathological (osteoarthritis and meniscal injury) and healthy knees.

**Methods:**

The Cochrane Controlled Register of Trials, PubMed, Medline, and Embase were used to perform a systematic review using the Preferred Reporting Items for Systematic Reviews and Meta-Analyses (PRISMA) criteria. Data pertaining to intra- and interrater reliability of US in measuring meniscal extrusion (ME), its correlation with magnetic resonance imaging (MRI), and head-to-head comparison of potential factors to influence ME were included [loading versus unloading position; osteoarthritis (OA) or pathological menisci (PM) versus healthy knees; mild versus moderate–severe knee OA]. Pooled data were analyzed by random or fixed-effects models.

**Results:**

A total of 31 studies were included. Intraclass correlation coefficients (ICC) for intra- and interrater reliability were minimum 0.94 and 0.91, respectively. The correlation between US and MRI was (*r* = 0.76). US detected ME to be greater in the loaded position in all knees (healthy, *p* < 0.00001; OA, *p* < 0.00001; PM, *p* = 0.02). In all positions, US detected greater extrusion in OA (*p* < 0.0003) and PM knees (*p* = 0.006) compared with healthy controls. Furthermore, US revealed greater extrusion in moderate–severe OA knees (*p* < 0.00001).

**Conclusions:**

This systematic review suggests ultrasonography can play an important role in the measurement of meniscal extrusion, with results comparable to that of MRI. However, to what extent it can differentiate between physiological and pathological extrusion requires further investigation, with an absolute cutoff value yet to be determined. Nevertheless, it is an appropriate investigation to track the progression of disease in those with meniscal pathologies or osteoarthritis. Furthermore, it is a feasible investigation to evaluate the meniscal function following surgery.

*Level of evidence*: IV, Systematic review of level III–IV evidence.

**Supplementary Information:**

The online version contains supplementary material available at 10.1186/s43019-024-00236-3.

## Introduction

Meniscal extrusion is associated with meniscal tears and loss of meniscal substance [1–3], and is thought to be an independent predictor of tibiofemoral cartilage loss [4, 5]. They can also occur with progressive osteoarthritis (OA) [6–8] and contribute to the appearance of joint space narrowing [9]. Longitudinal studies with large series have found higher baseline meniscal extrusion in either healthy knees or knees with OA to be an important indicator for the development of progressive OA changes in the following years [10–12]. Therefore, in this context, if one can identify pathological extrusion of the meniscus (> 3 mm) early on [1], it may become useful in selecting the appropriate treatment to prevent chondral injury or its progression. Magnetic resonance imaging (MRI) is widely accepted as the gold standard imaging method in the assessment of meniscal abnormalities including extrusion [4, 13]. However, this imaging is often time consuming, expensive, and not readily available. Furthermore, extrusion can often be occult and obscured, particularly in the supine non-weight-bearing positions to which MRI scans are generally performed under. As a result, the imaging does not consider the dynamic changes in the meniscus and thus may not be suitable for illustrating its configuration under load [14, 15].

The role of ultrasound (US) in orthopedics is rapidly evolving [16, 17]. There is growing evidence that it can evaluate meniscal extrusion with the knee in different positions, including axially loaded position [18–20]. Therefore, it may help to reveal extrusion that is not visible in conventional MRI scans, providing valuable information about the status of the meniscus or the progress of OA, and at a lower cost than MRI [21, 22]. However, as this is an emerging technique, the evidence for its use is uncertain. The purpose of this systematic review was twofold: (1) to synthesize the literature regarding the validity and reliability of US in diagnosing meniscal extrusion and (2) to recognize the extent of its value in assessing and detecting changes in meniscal extrusion under different load and articular cartilage conditions. In doing so, further context on its application in clinical use can be determined.

## Methods

### Literature search

A systematic review and meta-analysis were performed and reported according to the standards of the Preferred Reporting Items for Systematic Review and Meta-Analyses (PRISMA) criteria [23]. Searches of Cochrane Controlled Register of Trials, PubMed, Medline, and Embase were conducted from the inception of the databases to 28 December 2023. The Boolean search terms included: (“ultrasound” OR “ultrasonography” OR “sonography”) AND (“meniscus extrusion” OR “meniscus displacement” OR “meniscus radial displacement”). No restrictions were made on language, and efforts were made to obtain translated versions of all included studies. Restrictions were not placed on the date of publication or the journal. All relevant articles and reviews were examined for further relevant citations.

### Eligibility criteria and outcome measures

#### a. Inclusion criteria

All clinical and radiological studies were included if the validity of ultrasound for the detection of meniscal extrusion was evaluated through its correlation with MRI, which was used as the reference standard. Furthermore, those that reported on the reliability of ultrasonography were included. This included studies that provided intraclass correlation coefficients (ICC) (intra- and interrater reliability) to determine the agreement between assessments on US using the absolute values of measurements of meniscal extrusion. In addition, studies that provided a head-to-head comparison of potential factors that can influence the degree of meniscal extrusion were included. This included the use of US in loaded [standing, full weight-bearing (FWB)] versus unloaded [supine, non-weight-bearing (NWB)] positions, osteoarthritis (OA) versus healthy knees, pathological menisci versus healthy knees, and between mild (grade 2) to moderate–severe (grades 3 or 4) OA using the Kellgren–Lawrence (K/L) system. Healthy knees were those without any cartilage, menisci, or other soft-tissue pathology. In view of these criteria, this meta-analysis analyzed previous studies which were of level I (randomized controlled trials) to level IV (cases series) evidence.

#### b. Exclusion criteria

Exclusion criteria included biomechanical and non-human studies, participants < 18 years of age, case reports, expert opinions, and technical tips and publications pertaining solely to the description of measuring meniscal extrusion under US.

### Study selection and the assessment of quality of studies

Two authors (authors 1 and 4) independently reviewed the titles and abstracts from the search results, after which potentially suitable papers were reviewed in full-paper format by each author independently. Those that met the eligibility criteria were chosen and any discrepancies highlighted and resolved by the senior authors (authors 5, 6, and 7). Following this, authors 1 and 4 independently assessed the quality of the included clinical studies using the modified Coleman methodology score (MCMS) with scores designated as follows: excellent (> 85), good (70–84), fair (55–69), and poor (< 55). These have been described in previous reports (Additional file 1) [24–26]. The risk of bias of the included clinical studies was assessed and reported by the same two authors in accordance with the risk of bias in nonrandomized studies of interventions tool [27]. Each item was judged according to high, moderate, low or unclear risk of bias. Studies were deemed to have the highest risk of bias if they scored a high or unclear risk of bias.

### Data synthesis and statistical analysis

Meniscal extrusion (in mm) was synthesized as the weighted mean and standard deviation adjusted for sample size. Review Manager (RevMan) software (version 5.4, Cochrane Training, London, UK) and Stata (Stata Statistical Software: Release 18. College Station, TX: StataCorp LLC) were used for data synthesis. The latter was particularly in the context for correlation, where the lack of normal distribution required the data to undergo Fisher’s *Z* transformation to ensure the analysis was independent of sampling variance and distribution. Odd ratios (ORs) were used for all dichotomous variables and mean differences (MD) for continuous parameters. Statistical heterogeneity was assessed using the *I*^2^ and the chi-squared result. A *p* < 0.1 and an *I*^2^ > 50% were considered suggestive of statistical heterogeneity, prompting a random-effects model. Otherwise, a fixed-effects model was used.

## Results

**Fig. 1:**
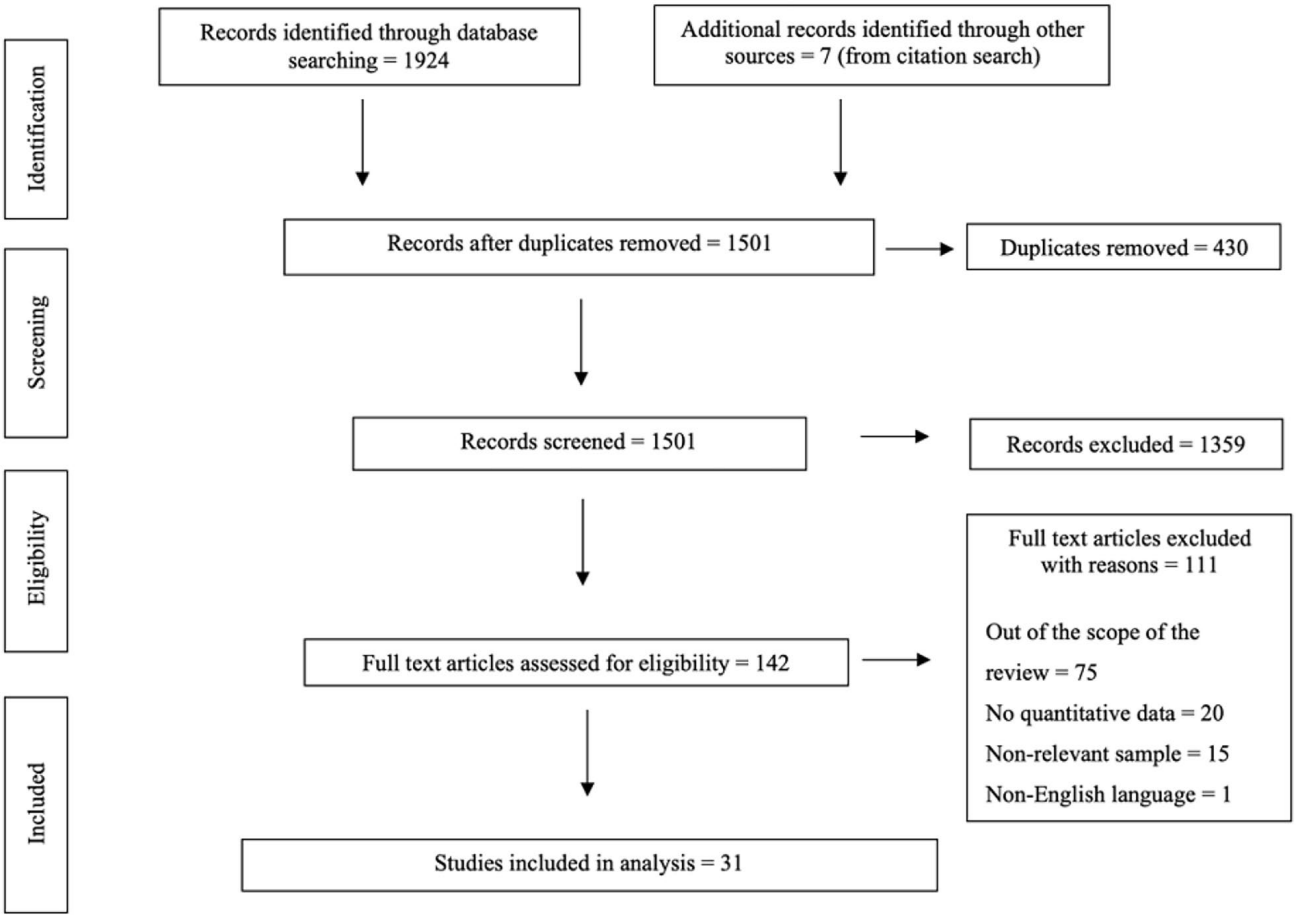
PRISMA flow diagram for study selection

A total of 1931 primary studies were identified before 430 duplicates were removed. After review of the titles and abstracts, 1359 studies were excluded, leaving 142 full-text articles for review. After screening for eligibility, only 31 papers met the criteria and were included (Fig. 1) [8, 19, 20, 28–55]

### a. Baseline characteristics

Regarding the Coleman methodology score, of the 31 included studies (mean score, 68.1), 1 study achieved an excellent score [34], 12 achieved good scores [29, 31, 38, 40, 42–45, 47, 49, 50, 53], 15 achieved fair scores [8, 19, 28, 30, 32, 33, 36, 37, 41, 46, 48, 51, 52, 54, 55], and 3 poor scores [20, 35, 39]. The overall quality of the studies was fair. The characteristics of these studies are described in Table 1.

**Table 1:** Baseline characteristics of included studies

Study	Study design (level of evidence)	Study description					Gender (m, %)	Age (mean ± SD)	BMI	MCMS	Value
		Patient knee cohort	Study size	Measurements of interest							
				Change (*Δ*) in meniscal extrusion between groups using US	Intraclass correlation for the reliability of US	Validation of US with MRI correlation					
Verdonk 2004 [20]	Prospective cohort (II)	10 LMA 10 healthy	10 (20 knees)	Yes: (1) ΔLME between supine (NWB) & UPS/BPS (FWB) position in both cohorts (2) ΔLME between healthy & LMA knees in all positions	Yes: Intrarater reliability for ΔLME using US in all positions for all knees	No	NR	33.8 ± 6.75	NR	50	Poor
Ko 2007 [8]	Prospective cohort (II)	97 healthy 141 OA (KL 1–2: 85, 3–4:56)	238	Yes: (1) ΔMME between healthy & OA knees, and between K/L grades in standing (FWB) position	NI	NI	94 (39.5)	60.5 ± 13.1	NR	66	Fair
Iagnocco 2012 [35]	Prospective case series (IV)	OA K/L unknown	9 (17 knees)	NI	Yes: Interrater reliability for ΔMME using US in supine NWB position	NI	2 (22.2)	61.33 ± 7.35	27.5 ± 4.3	50	Poor
Kawaguchi 2012 [19]	Prospective cohort (II)	20 healthy 78 OA (K/L 1:25, 2:33, 3:14, 4:6)	98	Yes: (1) ΔMME between supine (NWB) & standing (FWB) position in both cohorts (2) ΔMME between healthy & OA knees, and between K/L grades in all positions	Yes: Intrarater reliability for ΔMME using US in all positions for all knees	NI	30 (30.6)	66 ± 9.2	NR	60	Fair
Acebes 2013 [28]	Prospective cohort (II)	33 OA (K/L unknown) 13 healthy	32 (46 knees)	Yes: (1) ΔMME between supine (NWB), UPS pre- and post-walk (FWB) positions in both cohorts (2) ΔMME between healthy and OA knees in all positions	Yes. Intra- and interrater reliability for ΔMME using US in all positions for all knees	NI	5 (10.9)	63.6 ± 8.1	27.9 ± 5.3	63	Fair
Yanagisawa 2014 [53]	Prospective cohort (II)	44 healthy 87 OA (K/L 2:27, 3:30, 4:30)	81 (131 knees)	Yes: (1) ΔMME between supine (NWB) and standing (FWB) positions in both cohorts (2) ΔMME between healthy and OA knees, and between K/L grades in all positions	Yes: Intrarater reliability for ΔMME using US in all positions for all knees	NI	27 (33.3)	62.8 ± 11.5	NR	81	Good
Yanagisawa 2014* [54]	Prospective case series (IV)	83 knee pain 719 healthy	401 (802 knees)	NI	Yes: Intrarater reliability for ΔMME using US in standing FWB position for all knees	NI	149 (37.2)	63.5 ± 12.5	23.7 ± 3.0	69	Fair
Nogueira-Barbosa 2015 [42]	Prospective case series (IV)	Chronic pain	93	NI	Yes: Intra- and interrater reliability for ΔMME using US in supine NWB position	Yes: For MME in supine NWB position	50 (53.8)	41.5 ± 13.8	28.7 ± 5.8	78	Good
Yanagisawa 2015 [52]	Prospective cohort (II)	299 healthy 151 OA (K/L unknown)	225 (450 knees)	Yes: (1) ΔMME between supine (NWB) & standing (FWB) positions in both cohorts (2) ΔMME between healthy and OA knees in all positions	NI	NI	71 (15.8)	65.8 ± 9.4	NR	69	Fair
Podlipska 2016 [45]	Prospective cohort (II)	79 OA (K/L 0:2, 1:21, 2:19, 3:20, 4:17) 80 healthy	159	NI	Yes: Intrarater reliability for ΔMME using US in supine NWB position for all knees	No	60 (37.7)	57.7 ± 11.4	27.0 ± 4.3	80	Good
Razek 2016 [46]	Prospective case series (IV)	OA (K/L unknown)	80	NI	Yes: Interrater reliability for ΔMME using US in supine NWB position	NI	24 (30)	NR	NR	60	Fair
Chiba 2017 [31]	Prospective case series (IV)	OA (KL ≥ 2)	270 (460 knees)	NI	Yes: Interrater reliability for ΔMME using US in supine NWB position	NI	70 (25.9)	60.5 ± 10.8	23.5 ± 3.5	79	Good
Ishii 2017 [36]	Prospective cohort (II)	22 healthy 31 OA (K/L 2–4)	29 (53 knees)	Yes: (1) ΔMME between supine (NWB) & standing (FWB) positions in both cohorts (2) ΔMME between healthy and OA knees in all positions	NI	NI	5 (17.2)	72.3 ± 6.9	24.0 ± 3.0	63	Fair
Murakami 2017 [41]	Prospective cohort (II)	32 K/L 1 14 K/L 2	46	Yes: (1) ΔMME between supine (NWB) and standing (FWB) positions in both cohorts (2) ΔMME between K/L grades in all positions	Yes: Intra- and interrater reliability for ΔMME using US in all positions for all knees	NI	16 (34.8)	70.6 ± 2.8	24.1 ± 2.9	65	Fair
Achtnich 2018 [29]	Prospective case series (IV)	Healthy	75	Yes: (1) ΔMME between supine (NWB) and standing (FWB) positions	Yes: Intrarater reliability for ΔMME using US in all positions	No	17 (22.7)	39.6 ± 13.5	23.6 ± 3.9	71	Good
Diermeier 2019 [32]	Prospective case series (IV)	Healthy athletes	18	Yes: (1) ΔMME between supine (NWB) and standing (FWB) position in healthy knees (2) ΔMME between marathon stages in all positions	NI	No	13 (72.2)	37.4 ± 8.3	21.4 ± 1.2	67	Fair
Karpinski 2019 [40]	Prospective cohort (II)	25 Root tear 25 healthy	50	Yes: (1) ΔMME between supine (NWB) and standing (FWB) position in both cohorts (2) ΔMME between MT and non-MT groups in all positions	NI	No	27 (54)	57.8 ± 6.8	26 ± 3.3	72	Good
Ozdemir 2019 [44]	Prospective cohort (II)	91 OA (K/L 1:29, 2:34, 3:20, 4:8) 11 healthy	102	Yes: (1) ΔMME between supine (NWB) and standing (FWB) positions in both cohorts (2) ΔMME between healthy and OA knees, and between K/L grades in all positions	NI	No	44 (43.1)	48.1 ± 11.3	28.8 ± 5.7	72	Good
Elkwesny 2020 [33]	Prospective case series (IV)	OA with MMPRT (K/L unknown)	30	Yes: (1) ΔMME between supine (NWB) and standing (FWB) position	NI	No	6 (20)	47.8 ± 7.58	NR	59	Fair
Ishii 2020 [37]	Prospective cohort (II)	23 K/L 2 21 K/L 3–4	44	Yes: (1) ΔMME between supine (NWB) and UPS (FWB) positions in both cohorts (2) ΔMME between KL grades in all positions	Yes: Intrarater reliability for ΔMME using US in all positions for all knees	NI	22 (50)	68.9 ± 9.6	25.1 ± 3.1	58	Fair
Ishii 2020* [39]	Prospective cohort (II)	6 Healthy 6 OA (K/L 2–3)	12	Yes: (1) ΔMME between OA and healthy knees in standing FWB position	Yes: Intrarater reliability for ΔMME using US in standing FWB position for all knees	NI	8 (75)	46.8	22.9	54	Poor
Reisner 2020 [47]	Prospective cohort (II)	48 healthy 42 OA (K/L unknown)	45 (90 knees)	NI	Yes: Intra- and interrater reliability for ΔMME using US in all positions for all knees	NI	16 (35.6)	45 ± 5.46	27.0 ± 3.8	74	Good
Shimozaki 2020 [50]	Prospective case series (IV)	Healthy	18	Yes: (1) ΔMME between supine (NWB) & DLU/SLU (FWB) position	NI	Yes: For MME in all positions	13 (72.2)	21.8 ± 3.1	21.1 ± 1.7	78	Good
Cho JC 2021 [30]	Prospective case series (IV)	Healthy	35 (60 knees)	Yes: (1) ΔMME between supine (NWB), standing (FWB), and Thessaly (FWB) positions	NI	NI	Not reported	29 ± 4.5	NR	63	Fair
Reisner 2021 [48]	Prospective cohort (II)	48 healthy 42 medial OA	45 (90 knees)	Yes: (1) ΔMME between supine (NWB) and standing (FWB) positions in both cohorts (2) ΔMME between healthy and OA knees in all positions	NI	NI	16 (35.6)	45 ± 5.46	27 ± 3.8	63	Fair
Shimozaki 2021 [49]	Prospective case series (II)	Pre-OA knees (KL 0/1)	100	NI	Yes: Intrarater reliability for ΔMME using US in 0° and 90° flexion for all knees	Yes: For MME in supine NWB position	39 (39)	64.3 ± 7.9	NR	74	Good
Winkler 2021 [51]	Prospective cohort (II)	32 healthy 10 ACLR and LM radial repair	21 (42 knees)	Yes: (1) ΔLME between supine (NWB) and standing (FWB) position in both cohorts	Yes: Intra- and interrater reliability for ΔLME using US in all positions for all knees	Yes: For LME in all positions for all knees	17. (81.0)	27.6 ± 5.9	NR	67	Fair
Zeitoun 2021 [55]	Prospective cohort (II)	45 MT 58 healthy	103	Yes: (1) ΔMME between supine (NWB) and standing (FWB) position in both cohorts (2) ΔMME between MT and non-MT groups in all positions	NI	No	55 (53.4)	36.82 ± 13	NR	69	Fair
Falkowski 2022 [34]	Prospective cohort (II)	36 healthy 20 MD 43 MT	95 (99 knees)	Yes: (1) ΔMME between supine (NWB) and standing (FWB) positions in both cohorts (2) ΔMME between healthy, MT and MD knees in all positions on US	Yes: Interrater reliability for ΔMME using US in all positions for all knees	Yes: For MME in supine NWB position for all knees	50 (52.6)	45 ± 15	NR	91	Excellent
Oo 2022 [43]	Prospective case series (III)	Patients from RESTORE trial	89	NI	Yes: Interrater reliability for ΔMME using US in supine NWB position	Yes: For MME and LME in supine NWB position	41 (46.1)	61.5 ± 6.9	27.5 ± 6.4	74	Good
Ishii 2023 [38]	Prospective case series (IV)	Primary OA (K/L 1: 4, 2:17, 3:11)	32	Yes: (1) ΔMME between supine (NWB) and dynamic walking (FWB) positions	NI	No	13 (40.6)	60.5 ± 9.9	24.4 ± 3.2	71	Good

*ACLR* anterior cruciate ligament reconstruction, *BPS* bipedal stance, *DLU* double leg upright, *FWB* full-weight bear, *LM* lateral meniscus, *LMA* lateral meniscus allograft, *MD* meniscus deficient, *MMPRT* medial meniscus posterior root tear, *MT* meniscal tear, *NI* not investigated, *NR* not reported, *NWB* non-weight bear, *SLU* single leg upright, *UPS* unipedal stance

A total of 2685 individuals (1640 female, 1000 male, and 45 unknown) and 3747 knees, with a weighted mean age of 56.9 ± 10.6 years old and a weighted mean BMI of 25.2 ± 3.9 kg/m^2^ were included in this systematic review. Of these, 2046 knees were healthy, 1548 had OA, 133 had meniscal pathology (meniscal tear, meniscal root tear, degenerative changes), and 20 underwent lateral meniscal surgery to include either meniscal allograft transplantation (MAT) or meniscal repair. Medial meniscal extrusion (MME) was measured in 28 studies [8, 19, 28–42, 44–50, 52–55], with 2 studies measuring lateral meniscal extrusion (LME) [20, 51], and 1 measuring both [43]. In view of this, a pooled overall estimate on meniscal extrusion was provided; 19 studies measured intraclass correlation coefficients for intrarater reliability [19, 20, 29, 37, 39, 45, 49, 53, 54], interrater reliability [31, 34, 35, 43, 46], or both [28, 41, 42, 47, 51]. Rater reliability was deemed poor if the ICC was below 0.50, moderate if between 0.50 and 0.75, good if between 0.75 and 0.90, and excellent if above 0.90 [56].

A total of 6 studies assessed the validation of US for medial or lateral meniscal extrusion, through its correlation with MRI [34, 42, 43, 49–51]. The strength of the correlation was deemed very weak if *r* was < 0.20, weak if 0.20–0.39, moderate if 0.40–0.59, strong if 0.60–0.79, and very strong if > 0.80 [57]. Only 1 study documented extrusion to be measured at the same position on both US and MRI [42], and 20 studies assessed the value of US in both unloaded (supine/NWB) and loaded (standing/FWB) positions in evaluating the degree of meniscal extrusion [19, 20, 28–30, 32–34, 36–38, 40, 41, 44, 48, 50–53, 55]. All but 3 studies were on healthy knees [33, 37, 38], 11 on knees with OA [19, 28, 33, 36–38, 41, 44, 48, 52, 53], 3 on knees with pathological menisci [34, 40, 55], and 2 following meniscal surgery [20, 51].

A total of 14 studies assessed the effectiveness of US on potential factors that can influence extrusion in comparison with healthy knees. Of these, ten were knees with OA [8, 19, 28, 36, 39, 41, 44, 48, 52, 53], three in knees with meniscal injury [34, 40, 55], and one with meniscal transplantation [20]. Furthermore, five studies analyzed its use in detecting differences in meniscal extrusion on the basis of the severity of OA [8, 19, 37, 44, 53].

To conduct a meta-analysis, it was ensured that outcome measures of interest were measured in ≥ 3 studies, with precise means and similar metrics. Furthermore, in consideration of the dynamic changes of the meniscus upon loading,^24,36,40^ data on the position of the knee (i.e., supine or standing) were unpooled to minimize heterogeneity.

### b. Ultrasound assessment

The vast majority of studies displayed homogeneity in the performance and assessment of extrusion with US (Table 2). In particular, meniscal extrusion measurement was either the distance (mm) between the outermost edge of the meniscus and a vertical line connecting the femoral and tibial cortices [19, 20, 31, 33, 41, 43, 44, 47, 48, 52–55], or between the border of the tibial plateau and the outermost edge of the meniscal body [8, 29, 30, 32, 34, 36–40, 42, 49, 50]. Furthermore, US was generally performed in full knee extension in both supine and standing positions. Where described, the performance and analysis of US was undertaken by orthopedic surgeons or musculoskeletal radiologists, ranging from 3 to 23 years of US experience.

**Table 2:** Ultrasound assessment for meniscal extrusion

Study	How the meniscus was identified on imaging		Measurement of extrusion (mm)			Measured in full knee extension	Performance (P) and analysis (A)
	For medial meniscus, was longitudinal section of imaging parallel to the MCL used as reference?	For lateral meniscus	Medial meniscus extrusion		Lateral meniscus extrusion		
			Was extrusion measured between the outer edge of the MM and (a) a line connecting the femoral and tibial cortices or (b) the medial tibial border	Site of measurement on meniscus (meniscus body, anterior or posterior horn)			
Verdonk 2004 [20]	N/A	Probe placed anterior to LCL	N/A	N/A	Line between red and white zone and line connecting femoral and tibial cortex	Yes	P & A: physician of unknown experience
Ko 2007 [8]	Yes	N/A	Yes (b)	NR	N/A	Yes	P & A: physician of unknown experience
Iagnocco 2012 [35]	NR	N/A	NR	NR	N/A	No: Supine with 30° flexion	P & A by three sonographers of different levels (3 months, 5 years, and 24 years of experience)
Kawaguchi 2012 [19]	Yes	N/A	Yes (a)	NR	N/A	Yes	P & A by physician of unknown experience
Acebes 2013 [28]	Yes	N/A	No, between MCL and line connecting femur and tibial cortices	NR	N/A	Yes	P: unknown A: 2 rheumatologists
Yanagisawa 2014 [53]	Yes	N/A	Yes (a)	NR	N/A	Yes	P & A by experienced orthopedic surgeon
Yanagisawa 2014* [54]	Yes	N/A	Yes (a)	NR	N/A	Yes	P: orthopedic surgeon A: by physician of unknown experience
Nogueira-Barbosa 2015 [42]	Yes	N/A	Yes (b)	Meniscal body	N/A	Yes	P: MSK radiologist (3 years US experience) A: two MSK radiologists (3 and 8 years of experience)
Yanagisawa 2015 [52]	Yes	N/A	Yes (a)	NR	N/A	Yes	P & A by experienced orthopedic surgeon
Podlipska 2016 [45]	NR	N/A	NR	NR	N/A	Yes	P: Sonographer (2 days of training). A: unknown
Razek 2016 [46]	No, placed medial joint space	N/A	Inadequate description	NR	N/A	Yes	P: 1 MSK radiologist (20 years of experience) A: one radiologist and one rheumatologist (20 and 10 years of experience, respectively)
Chiba 2017 [31]	Yes	N/A	Yes (a)	NR	N/A	Yes	P & A: two orthopedic surgeons (3 and 6 years of experience, respectively)
Ishii 2017 [36]	No, placed at MCL and MM border	N/A	Yes (b)	NR	N/A	Yes	P: unknown A: one orthopedic surgeon
Murakami 2017 [41]	Insufficient description	N/A	Yes (a)	NR	N/A	Yes	P & A: two orthopedic surgeons
Achtnich 2018 [29]	Yes	N/A	Yes (b)	Meniscal body	N/A	Supine (NWB): Full knee extension Loaded: 20° flexion	P: MSK radiologist (20 years of US experience) A: one orthopedic surgeon and one MSK radiologist
Diermeier 2019 [32]	Yes	N/A	Yes (b)	Meniscal body	N/A	No. 20° flexion	P: orthopedic surgeon (2 years of experience) A: one orthopedic surgeon
Karpinski 2019 [40]	Yes	N/A	Yes (b)	Meniscal body	N/A	Yes	P & A: unknown
Ozdemir 2019 [44]	Yes	N/A	Yes (a)	NR	N/A	Yes	P: one MSK radiologist (9 years of experience) A: one MSK radiologist (20 years of experience)
Elkwesny 2020 [33]	Yes	N/A	Yes (a)	NR	N/A	Yes	P: unknown A: one MSK radiologist (12 years of experience)
Ishii 2020 [37]	No, placed at MCL and MM border	N/A	Yes (b)	NR	N/A	Yes	P & A: physician of unknown experience
Ishii 2020* [39]	Yes	N/A	Yes (b)	NR	N/A	Yes	P & A: physician of unknown experience
Reisner et al. 2020 [47]	Insufficient description	N/A	Yes (a)	NR	N/A	Yes	P & A by sports physician, sports fellow, and resident (30 min of experience of all)
Shimozaki 2020 [50]	Yes	N/A	Yes (b)	NR	N/A	Yes	P & A: experienced orthopedic surgeon
Cho JC 2021[30]	Yes	N/A	Yes (b)	NR	N/A	Yes	P & A: two chiropractors (1 and 10 years of US experience, respectively)
Reisner 2021 [48]	Insufficient description	N/A	Yes (a)	NR	N/A	Yes	P: ultra-sonographer (6 years of experience) A: unknown
Shimozaki 2021 [49]	Yes	N/A	Yes (b)	NR	N/A	No. at 0° and 90° degrees	P & A: one experienced orthopedic surgeon
Winkler 2021[51]	N/A	Probe between lateral femur and tibia condyle	N/A	N/A	Insufficient description	No. 10° knee flexion and 0° tibial rotation	P & A: two observers (unknown experience) and MSK radiologist
Zeitoun 2021[55]	Yes	N/A	Yes (a)	NR	N/A	Supine: 20–30° flexion Stand: Full extension	P & A: MSK radiologist (3 years of experience)
Falkowski 2022 [34]	Yes	N/A	Yes (b)	NR	N/A	Supine: Slight flexion Stand: full extension	P: MSK radiologist A: two MSK radiologists (7 and 23 years of experience)
Oo et al. 2022 [43]	Insufficient description	Insufficient description	Yes (a)	NR	Edge of lateral tibial plateau to most distant meniscus border	No. supine with 10° flexion	P & A by three observers: physician, MSK sonographer, and medical student (6 years, 8 years, and 1 week experience, respectively)
Ishii et al. 2023 [38]	Yes	N/A	Yes (b)	NR	N/A	Yes	P & A: physician of unknown experience

*LCL* lateral collateral ligament, *MCL* medial collateral ligament, *MM* medial meniscus, *MSK* musculoskeletal, *N/A* not applicable, *N/R* not reported

### c. Study risk of bias assessment

**Fig. 2:**
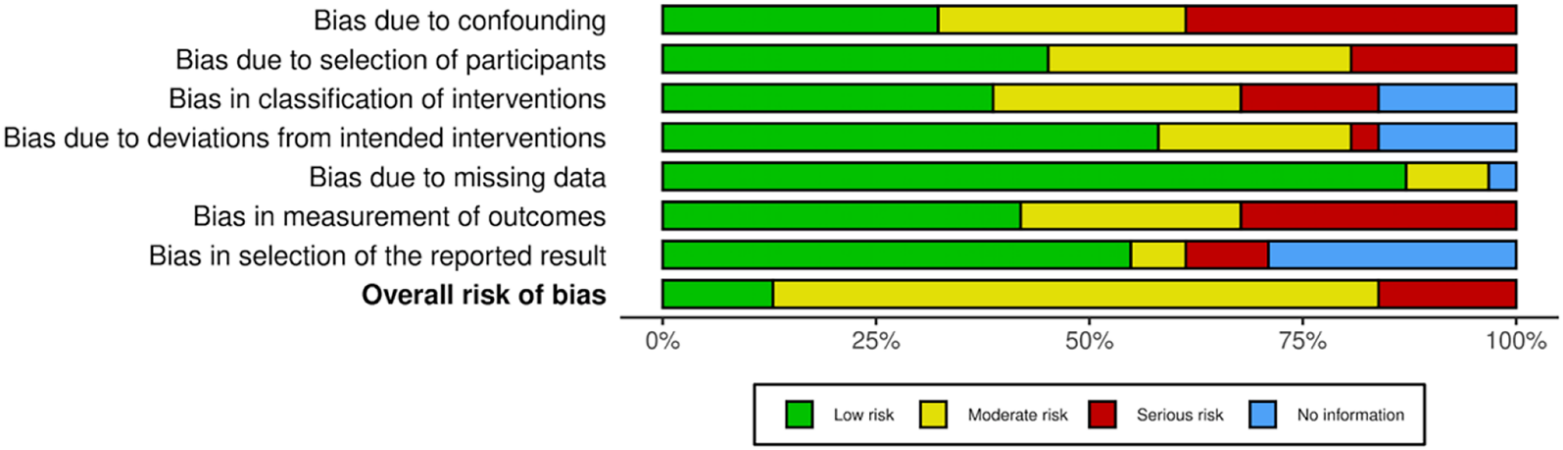
Risk of bias graph

Given the non-randomized design of the studies, the risk of selection and confounding bias was moderate to high (Table 3). Detection bias was similar, with evaluators who measured meniscal extrusion unblinded to the clinical data. The risk of attrition bias was low, and given there was no standardized protocol, several studies were judged as an unclear risk of reporting bias. The overall risk of bias was moderate. The Cochrane risk of bias graph is shown in Fig. 2.

**Table 3:**
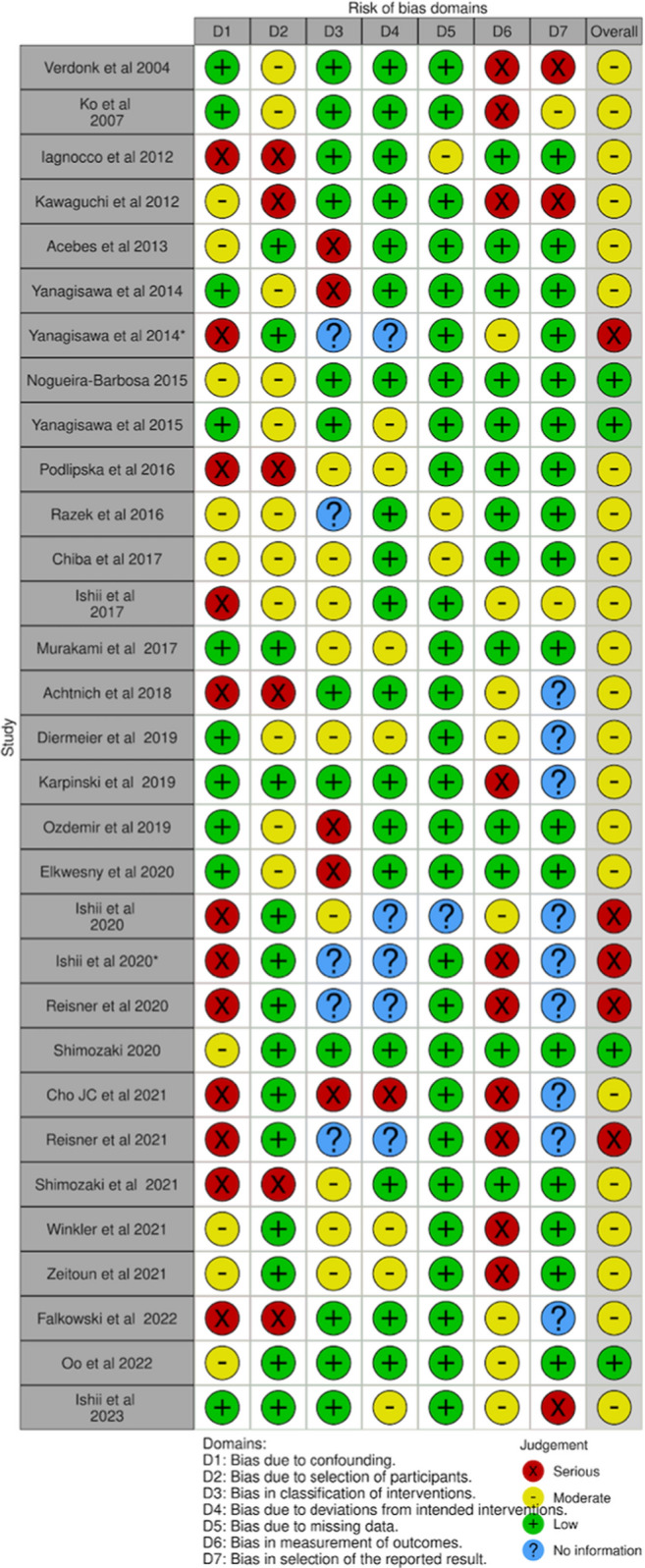
Risk of bias summary

Red circle, high risk of bias; yellow circle, moderate risk of bias; green circle, low risk of bias. D1: bias due to confounding data (selection bias), D2: bias in selection of participants into the study (selection bias), D3: bias in classification of interventions (information bias), D4: bias due to deviations from intended interventions (performance bias), D5: bias due to missing data (attrition data), D6: bias in measurement of outcomes (detection bias), and D7: bias in selection of the reported result (outcome reporting bias)

### d. Reliability and validity of US

Data on the reliability and validity of US in the detection and measurement of meniscal extrusion in supine and standing positions are presented as forest plots. Qualitative data can be found in additional file 2.

#### Reliability

**Fig. 3:**
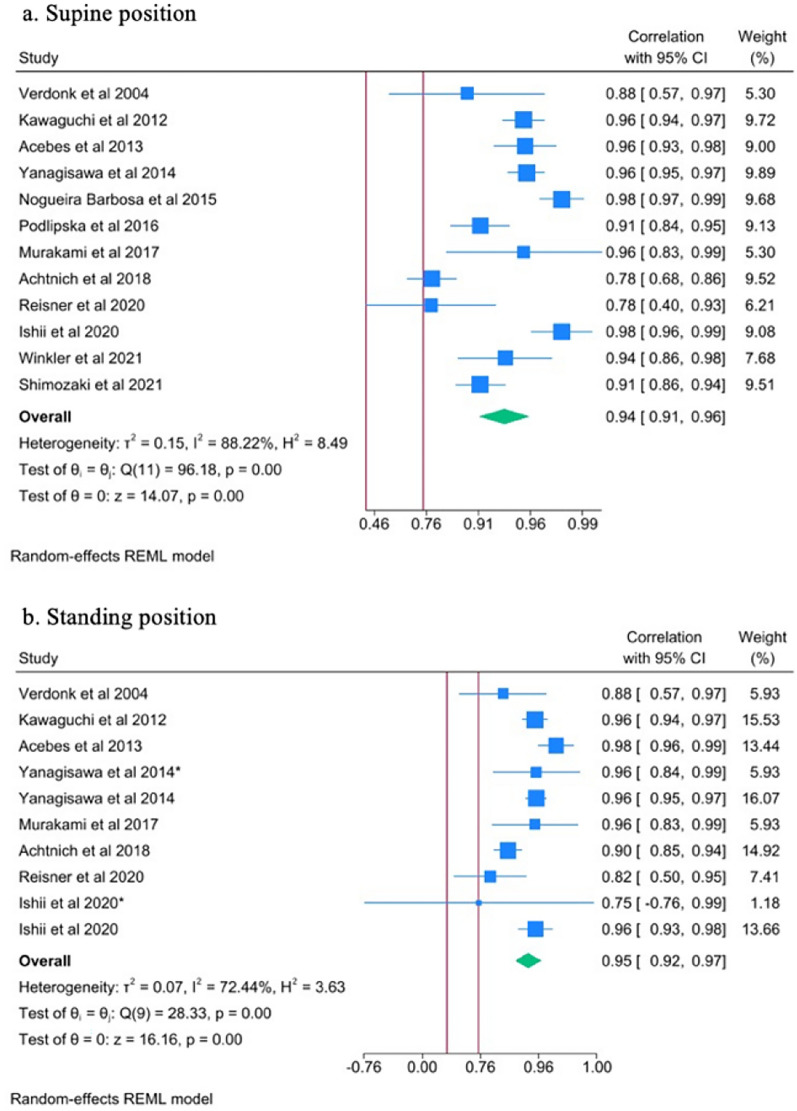
Forest plots of the intrarater reader reliability in diagnosing meniscal extrusion in supine and standing position of the knee. *, denotes a second independent paper performed by the same author that year

**Fig. 4:**
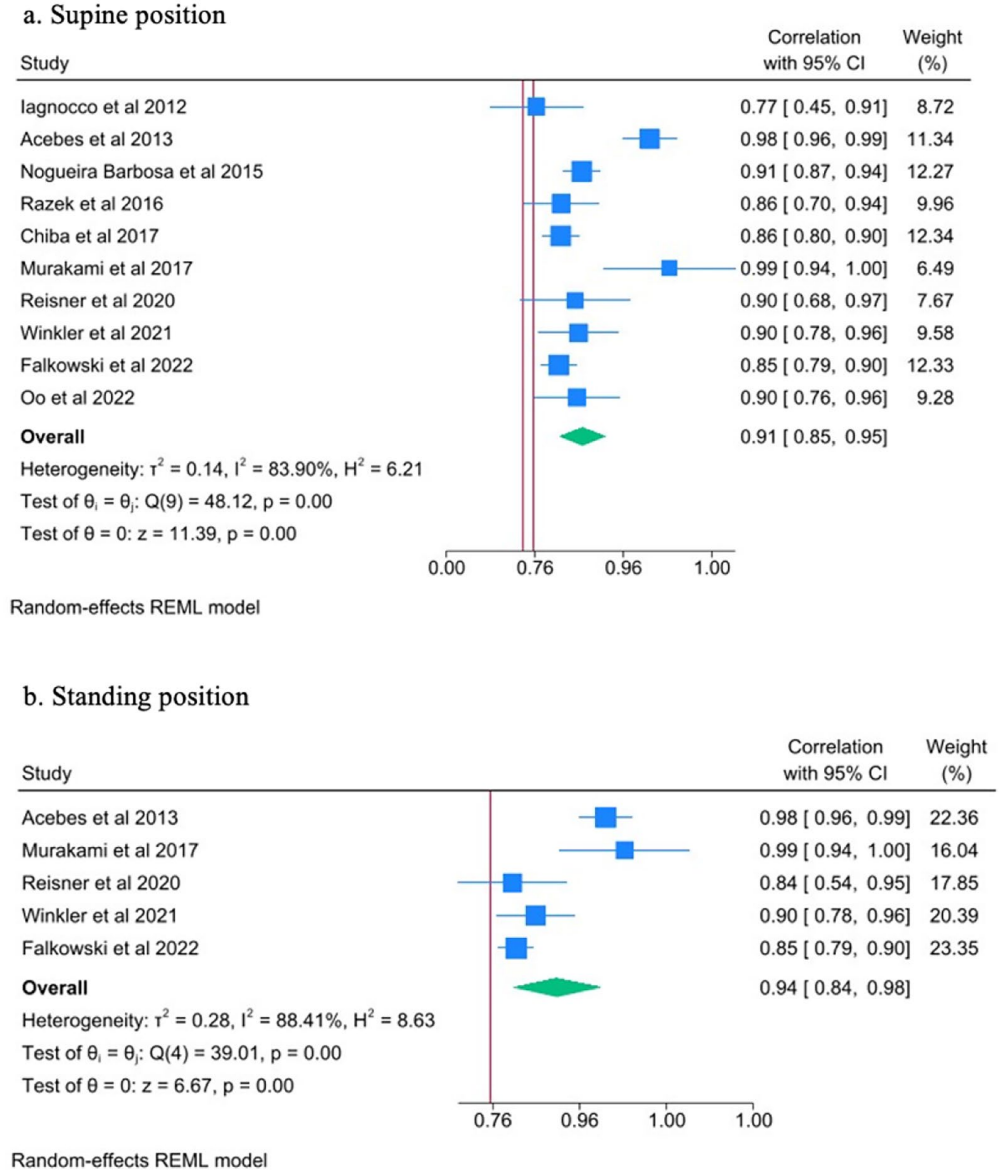
Forest plots of the interrater reader reliability in diagnosing meniscal extrusion in supine and standing position of the knee

For intrarater reliability, the ICC in both supine and standing position for all knees was 0.94 (95% CI 0.91–0.96) and 0.95 (95% CI 0.92–9.97), respectively (Fig. 3a, b). For interrater reliability, the ICC in both positions was 0.91 (95% CI 0.85–0.95) and 0.94 (95% CI 0.84–0.98) (Fig. 4a, b). These results are suggestive of excellent reliability of US in the measurement of meniscal extrusion.

#### Correlation to MRI

**Fig. 5:**
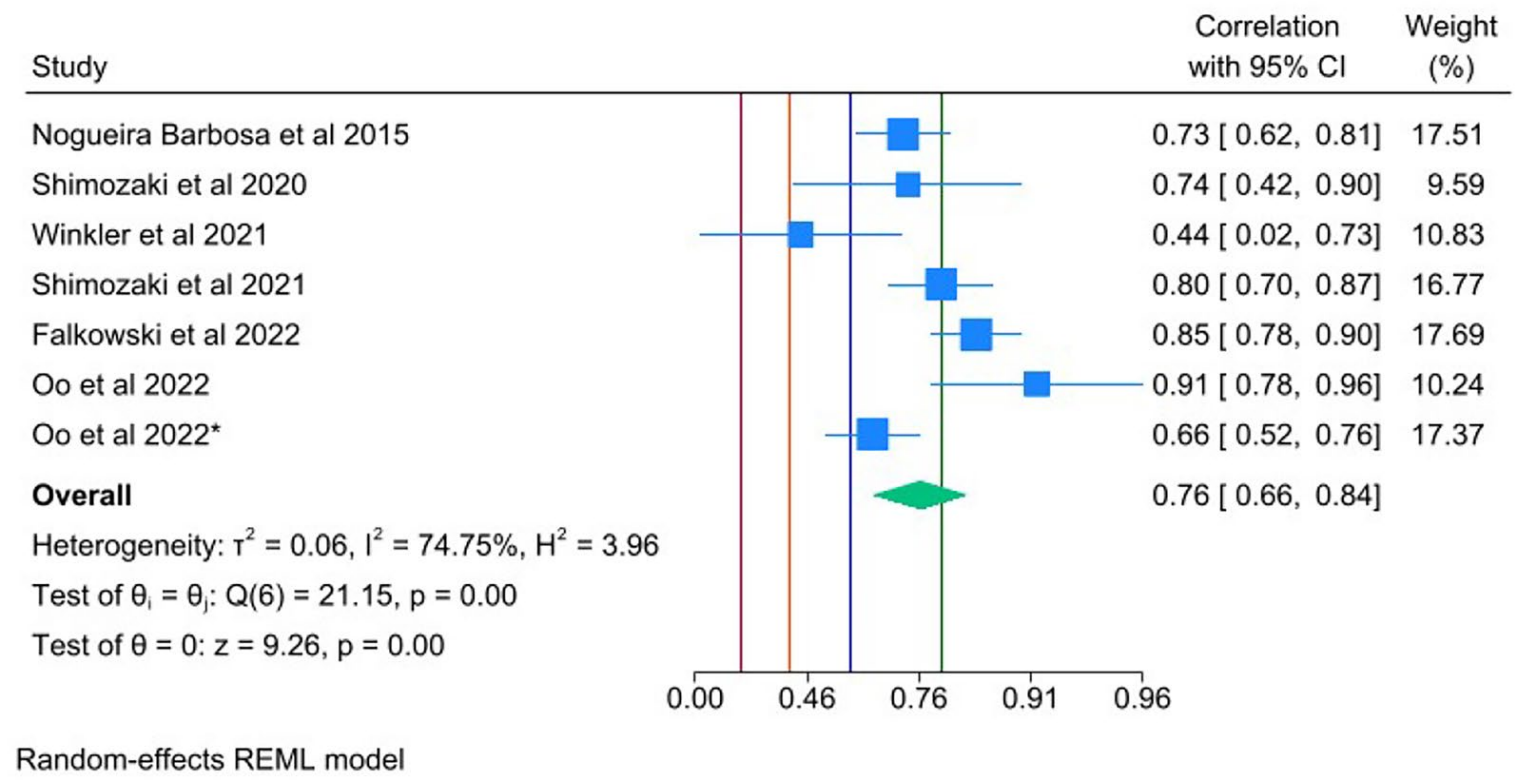
Forest plots of the correlation of US to MRI in supine position of the knee. Reference lines thresholds: 0.8, very strong (green); 0.6, strong (blue); 0.4, moderate (orange); 0.2, weak (red). *, denotes paper with separate date for medial and lateral meniscal extrusion

A total of six studies were included; four studies provided data on MME [34, 42, 49, 50], one on LME [51], and one on both [43]. Regarding the latter, the weighted mean for each outcome in this study was plotted separately. There was strong correlation between US and MRI in the measurement of meniscal extrusion in the supine position for all knees (0.76; 95% CI 0.66–0.84) (Fig. 5). Only two studies provided data in the standing position [50, 51], with moderate-to-strong correlation observed.

### e. Meniscal extrusion under load application

Data on the value of US in assessing changes in meniscal extrusion between standing (loaded) and supine (unloaded) positions are provided as forest plots. Qualitative synthesis can be found in additional file 3.

#### Healthy knees

**Fig. 6:**
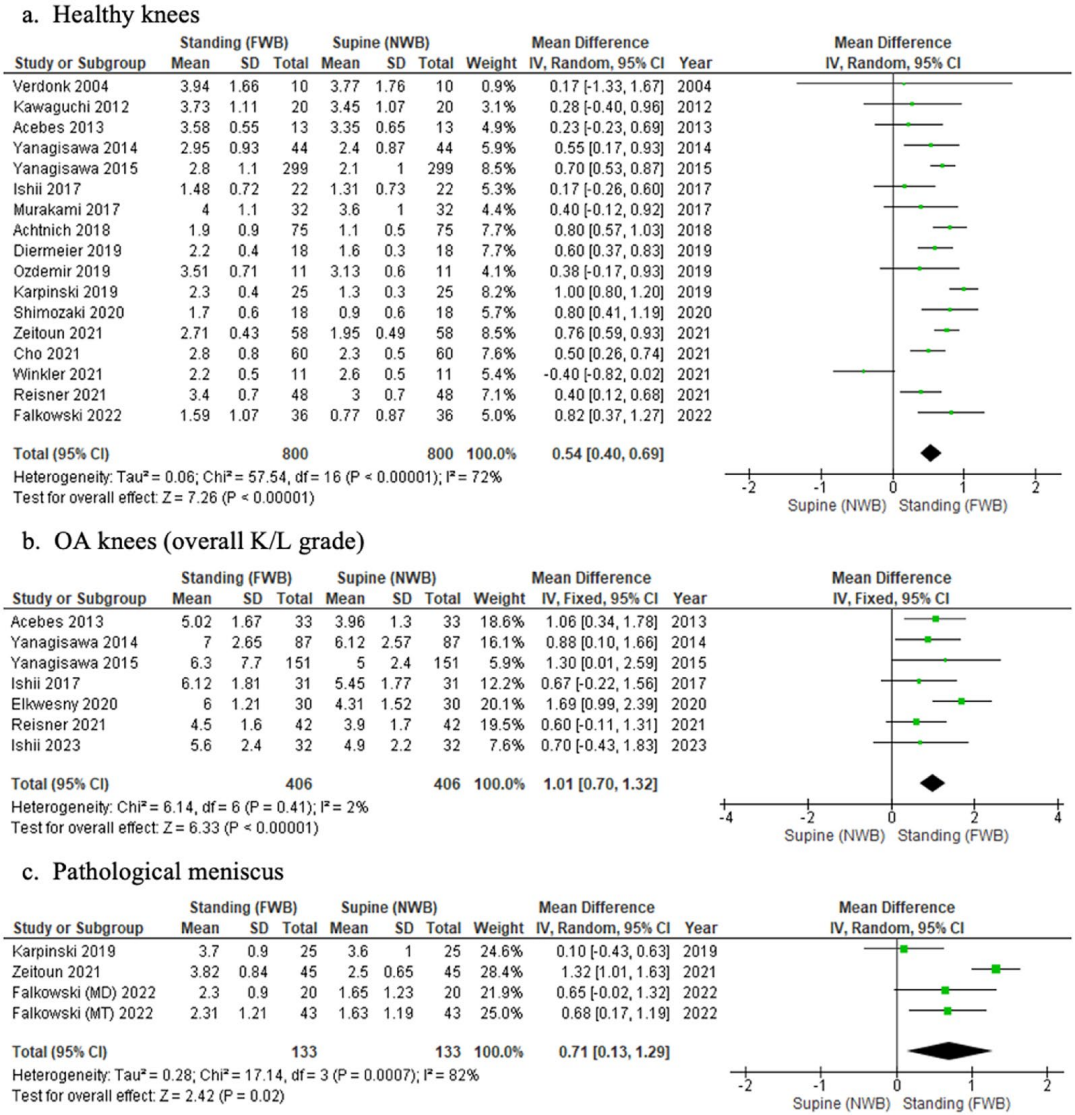
Forest plots of the change in meniscal extrusion between supine and standing positions in healthy, OA, and pathological menisci knees

US detected meniscal extrusion to be greater in the standing position (MD 0.54; 95% CI, 0.40–0.69) (*p* < 0.00001) (Fig. 6a).

#### OA knees

Only 7 of the 11 included studies provided a weighted mean across all K/L grades. In this case, US again detected meniscal extrusion to be greater in the standing position (MD 1.01; 95% CI 0.70–1.32) (*p* < 0.00001) (Fig. 6b). Of the remaining four studies [19, 39, 41, 44], individual K/L grades were provided with *p*-values described in three studies [19, 39, 41]. Qualitatively, US again revealed similar results.

#### Pathological menisci

Of the three studies [34, 40, 55] one study provided extrusion data for both meniscal tears and meniscal degeneration. The weighted mean for each outcome was plotted separately [34]. US detected greater extrusion in the standing position (MD 0.71; 95% CI 0.13–1.29) (*p* = 0.03) (Fig. 6c).

### f. Meniscal extrusion under various knee conditions

Data on the value of US in assessing changes in meniscal extrusion between pathological and healthy knees, and between K/L graded knees, in all positions, are presented as forest plots. Qualitative synthesis can be found in additional files 3 and 4.

#### Healthy versus OA knees

**Fig. 7:**
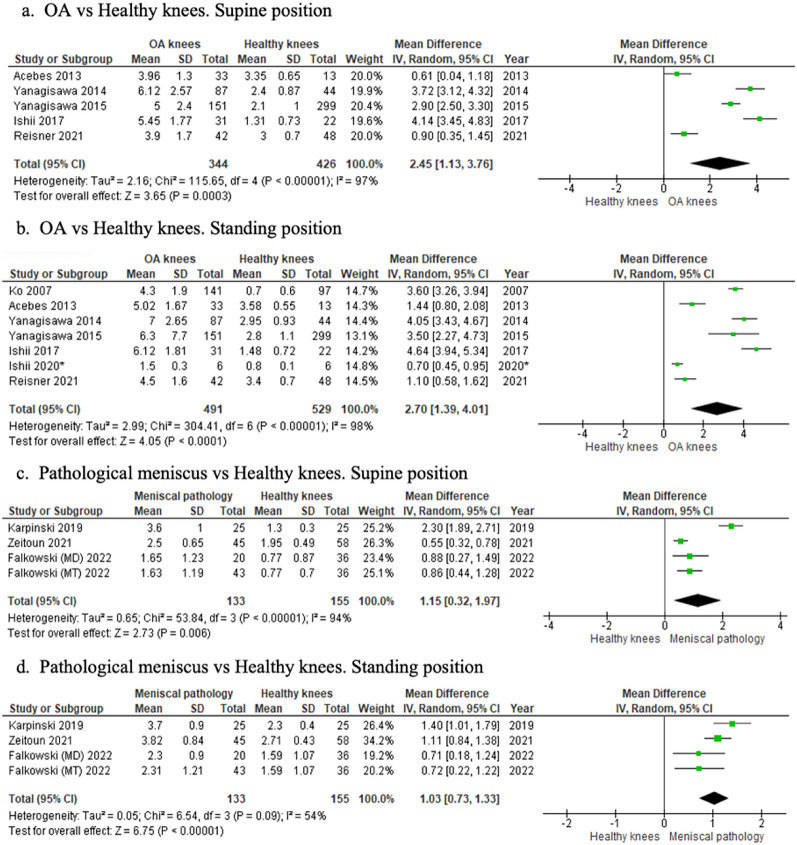
Forest plots of the change in meniscal extrusion between OA and healthy knees, and pathological menisci and healthy knees in all positions. *, denotes a second independent paper performed by the same author that year

US identified greater extrusion in the OA knees in both supine (MD 2.45; 95% CI 1.13–3.76) (*p* = 0.0003) and standing (MD 2.70; 95% CI 1.39–4.01) (*p* < 0.0001) positions (Fig. 7a, b). Qualitatively, studies on individual K/L grades found US detected greater extrusion in K/L ≥ 2 knees compared with their healthy counterparts [19, 41, 44].

#### Healthy versus pathological menisci

US detected greater extrusion in knees with meniscal injury in both supine (MD 1.15; 95% CI 0.32–1.97) (*p* = 0.006) and standing (MD 1.03; 95% CI 0.73–1.33) (*p* < 0.00001) positions (Fig. 7c, d).

#### K/L 2 (mild) versus K/L 4 (severe) OA

**Fig. 8:**
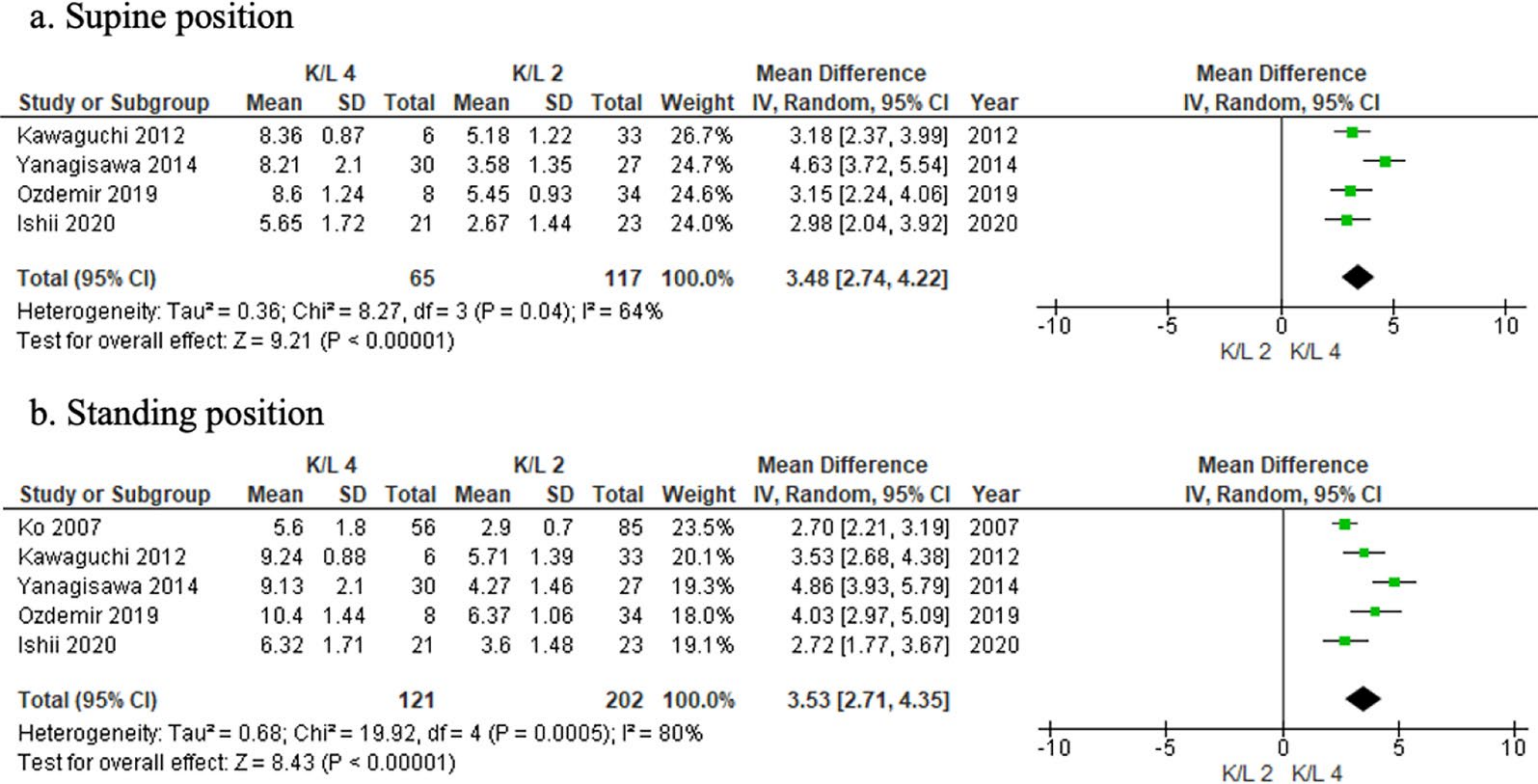
Forest plots of the change in meniscal extrusion between mild and moderate–severe OA using the K/L system in supine and standing positions

US revealed greater extrusion in patients with K/L 4 knees compared with those with K/L 2 in both supine (MD 3.48; 95% CI 2.74–4.22) and standing (MD 3.53; 95% CI 2.71–4.35) positions (Fig. 8a, b).

## Discussion

The present review indicates that US can both reliably measure meniscal extrusion and provide comparable results to that of MRI as the reference standard. Overall, extrusion increased significantly under load in all groups, suggesting the possibility that it may be obscured in unloaded positions. This meta-analysis indicates that the use of weight-bearing US could help in the identification of occult extrusion not visible on conventional MR imaging. Finally, it also suggests that US can delineate the extent of extrusion between those with advanced OA or meniscal tears, and those with healthy knees or mild osteoarthritis. In view of these findings, US is a feasibly acceptable and alternative tool in the dynamic assessment of meniscal extrusion, enabling further understanding of meniscus function compared with static imaging, all at lower costs with no radiation, the ability to repeat as often as necessary, and reducing the time to interventions.

Owing to the load-dependent extrusion in healthy controls in this review, extrusion should not always be regarded as a pathological finding [58, 59]. A certain degree of extrusion occurs with knee joint movement in the physiological state, owing to the viscoelastic properties of the meniscal tissue [60]. Axial load leads to a temporal limited, reversible deformation of the meniscus [7]. The collagenous fibers of the meniscus elongate and subsequently increase the hoop stress [29]. After unloading, the meniscus returns to its original position. Therefore, an increased meniscus extrusion in standing position can not only be regarded as pathologic, but a decreased or absent functional adaptation reaction of the meniscus can as well [29]. There is still no consensus with respect to the amount of meniscal extrusion that can be considered physiological, but authors have accepted up to 3 mm as normal [61, 62]. However, this systematic review revealed six studies on healthy cohorts to fall outside this physiological limit [19, 20, 28, 41, 44, 48] (see online resource 3 for qualitative data), and therefore, the current cutoff value of 3 mm should be reconsidered. Alternatively, it may be more appropriate that the decisive point be an absent or elevated extrusion under axial load. For example, a meniscus tear results in disruption of circumferential collagen fibers [63], and thus under load, the ability to resist hoop stresses is reduced. This phenomenon is less likely to occur without loading, thereby underestimating extrusion. Therefore, the extent of extrusion (difference between unloaded and loaded positions) that can be measured using US might be a better indicator for meniscus function compared with extrusion observed using static MRI. However, elevated extrusion under load can still occur with healthy knees. Therefore, even though greater levels of extrusion are detected by US in the pathological knees as earlier described, to what extent such elevation deems it pathological requires further investigation. From then, a new absolute cutoff value for physiological extrusion can be developed, or the alternative consideration of more stringent cutoff measures to include the absolute difference or ratio between the loaded and unloaded position.

Several studies suggest meniscal extrusion to be associated with OA and to worsen with its progression [3, 6, 7, 9]. This was reflected in this review where US detected greater extrusion in OA knees compared with healthy patients, and in those with moderate-to-severe OA. At present, there is no available evaluation method for predicting progression of knee OA. K/L grading is a plain radiological diagnosis and is commonly accepted by clinicians as a standard grading system for knee OA. However, the K/L grade cannot characterize chondral injury, including the meniscal injury as meniscal extrusion cannot be detected by radiography. Furthermore, MRI studies have shown an association between meniscal extrusion and early joint space narrowing [6, 64]. This lends support to the theory that early knee OA (KL 1 and 2) could be due to meniscal extrusion rather than cartilage thinning [9, 65], suggesting cartilage degeneration may well be a secondary event. Therefore, if the use of US is widely established, it may prove to be a valuable adjunct to radiographs, both for screening and estimating the severity of knee OA by simply measuring meniscal extrusion in supine and standing positions. Subsequently, early detection of meniscal extrusion may help prevent further deterioration of articular cartilage by selecting treatment designed in delaying or preventing its progress.

One way to assess the success of meniscus surgery to include meniscus transplantation and repair is the presence of extrusion [66]. Although MRI can play a role, variable signal intensities from scar tissue, revascularization, and meniscal tears can lead to suboptimal evaluation [67]. A total of two studies in this review revealed extrusion to be corrected following meniscus surgery [20, 51], raising the possibility of using weight-bearing US as a possible pre- and post-surgical scanning tool to follow up post-surgical outcome and longitudinally tracking the progression over time. This systematic review reflects the increasing importance of evaluating dynamic meniscal extrusion, and underlines the necessity to develop cheap, simple, and readily available alternatives to MRI for its assessment. Although US is operator dependent, with unclear anatomical landmarks, attaining the appropriate competency, both for its use and within a respectable timeframe, is feasible. This is underlined in this review through the vast homogeneity in the results of the measured outcomes by a range of healthcare professionals with varying training and experience.

To generate more meaningful intraclass correlation coefficients, one needs to obtain a heterogeneous cohort [68, 69]. This will facilitate the ability to determine both the true inter- and intrarater reliability and agreement with MRI as the reference standard. This was observed in several studies in this meta-analysis [19, 20, 28, 34, 37, 39, 41, 45, 51, 53, 54]. Although the majority of studies did not report on whether the position of the US for ME matched MRI, we speculate that the strong correlation observed suggests the effect of the position on the results were small.

This systematic review had several strengths, particularly in the homogeneity of the methodology of included studies. First, all studies with an adequate description of the US technique used the tibial edge as a reference point rather than the femur for meniscal extrusion measurement. Meniscal pseudo-subluxation, as described by Pomeranz et al. [70], is likely if the femoral condyle is used as the reference. In most cases, the position of the meniscus on the tibial plateau is stable and a true extrusion has not occurred. It is important that the tibial edge is used as the reference point for proper determination of meniscal position, as the meniscotibial attachment is stronger than that to the femur [71]. Second, most studies performed the US examination in the same position (full knee extension), enabling standardization across the board. However, no study examined anterior or posterior extrusion from the menisci respective horns. This was likely due to the inability of the US to reach the required depth. Despite this limitation in the US device, the likelihood of anterior or posterior extrusion is minimal. This is because in 40% of cases, the anterior horn does not attach to the tibial plateau but to the anterior surface of the tibia [72]. Furthermore, in full knee extension the medial meniscus is located in the anterior part of the tibial articular surface [73]. Therefore, significant anterior extrusion under loading may not occur. The posterior horn is closely coupled with the posterior capsule, meniscotibial ligament, and semi-membranosus tendon [73]. In knee extension, these tissues increase in tension, retaining the position of the posterior horn and thereby minimizing its extrusion.

### Limitations

There are several limitations of this review. All but one populated forest plots displayed high levels of heterogeneity (*I*^2^ > 50%). This may be due to several reasons. First, due to the differing levels of operator experience in assessing meniscal extrusion between studies, there was no standardized approach one could follow to narrow measurement error. This could possibly lead to under- or overestimation of extrusion values between studies. However, qualitative review of each individual study (online resources 2, 3, and 4) showed a similar trend between studies regarding the extent of extrusion in certain states and in its reliability and correlation to MRI. This therefore suggests ultrasonography to more likely have a true rather than exaggerated value in clinical practice in measuring extrusion. Second, the included studies were heterogeneous regarding the populations included, reflecting the high risk of selection bias due to uncontrolled confounding variables. However, several efforts were made to stratify into different categories (loaded versus unloaded, OA versus healthy, meniscal injury versus healthy, mild versus moderate–severe OA). Third, a recent biomechanical study states that the maximum von Mises stress varies depending on the OA state and the location of the measurement [74]. The latter underlines the importance of the site of measurement within the meniscus of each study included in the analysis. Only four studies described the exact location, all of which were on the meniscal body [29, 32, 40, 42]. Therefore, it is possible that the observed heterogeneity resulted from measurements made at other sites of the meniscus in the remaining studies.

Lower limb axis alignment was poorly reported, preventing a risk analysis on this characteristic. More than half of studies were judged as being moderate-to-high risk of detection bias, as the evaluators measuring meniscal extrusion were unblinded and may have influenced measurements of meniscal extrusion.

The majority of the studies performed US under static conditions, whereby the stress on the meniscus is different to that under dynamic conditions such as walking [75]. This might underestimate the reaction of the meniscus in daily activities. A dynamic approach may be a valid evaluation tool for truly measuring meniscal extrusion and understanding its pathological behaviour.

## Conclusions

This systematic review suggests ultrasonography can play an important role in the measurement of meniscal extrusion, with results comparable to that of MRI. However, to what extent it can differentiate between physiological and pathological extrusion requires further investigation, with an absolute cutoff value yet to be determined. Nevertheless, it is an appropriate investigation to track the progression of disease in those with meniscal pathologies or osteoarthritis. Furthermore, it is a feasible investigation to evaluate the meniscal function following surgery.

## Supplementary Information

Additional file1. Modified Coleman criteria used for assessment of the quality of clinical studies. Reliability of US and its correlation with MRI. The use of US in detecting changes in extrusion between both loading and unloading positions and different knee states. *MD* meniscal degeneration. The use of US in detecting changes in extrusion between mild (K/L 1 or 2) and moderate-severe (K/L 3 or 4) OA knees.

## Data Availability

The datasets generated during and/or analyzed during the current study are available from the corresponding author on reasonable request.

## Abbreviations

BMI: Body mass index
DLU: Double leg upright
FWB: Full weight bearing
ICC: Intraclass correlation co-efficient
K/L: Kellgren–Lawrence
LMA: Lateral meniscus allograft
LME: Lateral meniscus extrusion
MCMS: Modified Coleman methodology score
MD: Meniscus deficient
ME: Meniscal extrusion
MME: Medial meniscus extrusion
MMPRT: Medial meniscus posterior root tear
MRI: Magnetic resonance imaging
MSK: Musculoskeletal
MT: Meniscal tear
NI: Not investigated
NR: Not reported
NWB: Non-weight bearing
OA: Osteoarthritis
OR: Odds ratio
PM: Pathological menisci
PRISMA: Preferred Reporting Items for Systematic Reviews and Meta-Analyses
SLU: Single leg upright
SMD: Standard mean difference
UPS: Unipedal stance
US: Ultrasound
WB US: Weight-bearing ultrasound
