# Supplementary material for: Assessment of meniscal extrusion with ultrasonography: a systematic review and meta-analysis
Source: Knee Surg Relat Res. 2024 Oct 28;36:33. doi: 10.1186/s43019-024-00236-3 (PMC11514433; doi:10.1186/s43019-024-00236-3)
Supplement: Supplementary file 1 — Additional file1. Modified Coleman criteria used for assessment of the quality of clinical studies. Reliability of US and its correlation with MRI. The use of US in detecting changes in extrusion between both loading and unloading positions and different knee states. MD meniscal degeneration. The use of US in detecting changes in extrusion between mild (K/L 1 or 2) and moderate-severe (K/L 3 or 4) OA knees. [file 43019_2024_236_MOESM1_ESM.docx]

| Criteria | Domains | Score |
| --- | --- | --- |
| **Part A** |  |  |
| Study Size (total patients) | > 100 | 15 |
|  | 50 - 99 | 10 |
|  | 15 - 49 | 5 |
|  | < 15, not stated | 0 |
| Type of study (methodology) | Comparative | 12 |
|  | Non-comparative/case series | 6 |
| Diagnostic certainty (clinical examination, ultrasound, MRI) | In all | 5 |
|  | > 80% | 3 |
|  | < 80% | 0 |
| **Part B** |  |  |
| Outcome criteria (12) | Clearly defined outcome | 4 |
|  | PROMs used | 4 |
|  | Additional radiological investigation | 4 |
| Procedures for assessing outcome (12) | Clearly defined | 3 |
|  | Objective | 3 |
|  | Computer algorithm, blinding | 3 |
|  | Multiple observers | 3 |
| Description of subject population (10) | Inclusion criteria reported and unbiased | 4 |
|  | Recruitment rate reported  > 80% | 3 |
|  | Recruitment rate reported  < 80% | 2 |
|  | Recruitment rate not reported | 0 |
|  | All eligible subjects accounted for in methodology | 3 |
| Ultrasound Procedure (10) | Well described | 10 |
|  | Not adequately described | 5 |
|  | Inadequate, not stated or unclear | 0 |
| Measurement of Extrusion on US (8) | Well described | 8 |
|  | Not adequately described | 4 |
|  | Protocol not reported | 0 |
| Reference standard imaging (8) | Well described | 8 |
|  | Inadequately described | 4 |
|  | Not used | 0 |
| Assessment of other features on US* (8) | ≥ 2 features | 8 |
|  | 1 feature | 4 |
|  | No assessment | 0 |
|  |  |  |

**Additional file 1**. Modified Coleman Criteria used for Assessment of the Quality of Clinical Studies

^a^ The values in parenthesis are total cumulative points. * - to include effusion, osteophytes, cartilage thickness, synovial hypertrophy, bursitis, Baker’s cyst, meniscal tear

|  |  |  |  |
| --- | --- | --- | --- |
| Study | Inter-rater reliability of US  (n, number of knees) | Intra-rater reliability of US  (n, number of knees) | Correlation to MRI |
| Verdonk 2004 [20] | N/A | **Supine & Standing:**  Transplanted meniscus: 0.933, p < 0.001 **(n = 10)** Normal meniscus: 0.883, p < 0.001 **(n = 10)** | N/A |
| Iagnocco 2012 [35] | **Supine** 30° flexion: 0.767 **(n = 17)** | N/A | N/A |
| Kawaguchi 2012 [19] | N/A | **Supine and Standing:** 0.959, p < 0.001 **(n = 98)** | N/A |
| Acebes 2013 [28] | **Supine:** 0.98, p < 0.0001 **UPS before walking**: 0.98,  P < 0.0001 **UPS after walking**: 0.98, p < 0.0001 **(n = 46)** | **Supine**: 0.96, p < 0.0001 **UPS before walking**: 0.98, P < 0.0001 **UPS after walking**: 0.96, p < 0.0001  **(n = 46)** | N/A |
| Yanagisawa 2014 [53] | N/A | **Supine & Standing:** 0.961, p < 0.001 **(n = 131)** | N/A |
| Yanagisawa 2014* [54] | N/A | **Standing**: 0.961, p < 0.001 **(n = 10)** | N/A |
| Nogueira Barbosa 2015 [42] | **Supine**: 0.91  (95% CI, 0.66 - 1.0) (**n = 93)** | **Supine**: 0.98  (95% CI, 0.94 - 1.00) **(n = 93)** | **Supine**: r = 0.73 (95% CI, 0.58 - 0.79) |
| Podlipska 2016 [45] | N/A | **Supine**: 0.908, (95% CI, 0.845 - 0.947) **(n = 51)** | N/A |
| Razek 2016 [46] | **Supine**: 0.86 (95% CI, 0.73 - 0.99)  **(n = 25)** | N/A | N/A |
| Chiba 2017 [31] | **Supine**: 0.859 (95% CI, 0.668 - 0.944) **(n = 100)** | N/A | N/A |
| Murakami 2017 [41] | **Supine & Standing:**  0.987 (p < 0.001), **(n = 10)** | **Supine & Standing:**  0.959 (p < 0.001), **(n = 10)** | N/A |
| Achtnich 2018 [29] | N/A | **Supine**: 0.783 (95% CI, 0.625 - 0.872) **Standing**: 0.900 (95% CI, 0.820 - 0.942) **(n = 75)** | N/A |
| Ishii 2020 [37] | N/A | **Supine**: 0.98 (95% CI, 0.923 - 0.995) **Standing:** 0.96 (0.866 - 0.991) **(n = 49)** | N/A |
| Ishii 2020* [39] | N/A | **Standing**: 0.75 (95% CI, 0.169 - 0.98) **(n = 4)** | N/A |
| Reisner 2020 [47] | **Supine**: 0.896 (95% CI, 0.837 - 0.933) **Standing**: 0.842 (95% CI, 0.770 - 0.893) **(n = 13)** | **Supine**: 0.780 (95% CI, 0.664 - 0.856) **Standing**: 0.824 (95% CI, 0.731 - 0.884) **(n = 19)** | N/A |
| Shimozaki 2020 [50] | N/A | N/A | **Supine:** r = 0.74, **DLU**: r = 0.71,  **SLU**: r = 0.61 |
| Shimozaki 2021 [49] | N/A | 0° extension: 0.908 90° flexion = 0.898 **(n = 74)** | **Supine**: r = 0.8 |
| Winkler 2021[51] | **Supine & Standing:**  0.904 (95% CI, 0.824 - 0.947)  **(n = 22**) | **Supine**: 0.942 (95% CI, 0.861 - 0.976)  **(n = 22)** | **Supine & standing** 0.439 (95% CI, -0.221 - 0.750) |
| Falkowski 2022 [34] | **Supine**: 0.853 **Standing**: 0.885 **(n = 99)** | N/A | **Supine**: r = 0.85 |
| Oo 2022 [43] | **Supine**: 0.90, (95% CI, 0.75 to 0.96)  (**n = 20)** | N/A | **MME.** Supine: 0.91 (95% CI, 0.87 - 0.94) **LME.** Supine: 0.66 (95% CI, 0.52 - 0.76) |

**Additional file 2.** Reliability of US and its correlation with MRI.

|  |  |  |  |
| --- | --- | --- | --- |
|  | Change in ME (mm) with US | | |
| Study | Between loading conditions | Between meniscal states | Between healthy and OA knees |
| Verdonk 2004 [20] | **Healthy knees** Standing ~ Supine  3.94 ± 1.66 vs 3.77 ± 1.76, p < 0.05 **Lateral meniscal allograft** Standing ~ supine  6.14 ± 1.93 vs 6.43 ± 1.84, p > 0.05 | **Supine** LMA > Healthy  6.43 ± 1.84 vs 3.77 ± 1.76, p < 0.005 **Standing** LMA > Healthy 6.14 ± 1.93 vs 3.94 ± 1.66, p < 0.005 | N/A |
| Ko 2007 [8] | N/A | N/A | **Standing**  OA > healthy knees 4.3 ± 1.9 vs 0.7 ± 0.6, p < 0.001 |
| Kawaguchi 2012 [19] | **Healthy knees** Standing > Supine  3.73 ± 1.11 vs 3.45 ± 1.07 p < 0.05 **K/L 2:** Standing > supine 5.71 ± 1.39 vs 5.18 ± 1.22, p < 0.05 **K/L 3**: Standing > supine 7.76 ± 1.17 vs 6.83 ± 1.21, p < 0.05 **K/L 4**: Standing ~ Supine 9.24 ± 0.88 vs 8.36 ± 0.87, p = 0.06 | N/A | **Supine**  K/L 2 > healthy: 5.18 ± 1.22 vs 3.45 ± 1.07, p < 0.05 K/L 3 > healthy: 6.83 ± 1.21 vs 3.45 ± 1.07, p < 0.05 K/L 4 > healthy: 8.36 ± 0.87 vs 3.45 ± 1.07, p < 0.05 **Standing** K/L 2 > healthy: 5.71 ± 1.39 vs 3.73 ± 1.11, p < 0.05 K/L 3 > healthy: 7.76 ± 1.17 vs 3.73 ± 1.11, p < 0.05 K/L 4 > healthy: 9.24 ± 0.88 vs 3.73 ± 1.11, p < 0.05 |
| Acebes 2013 [28] | **Healthy knee** Standing pre-walk ~ Supine   3.58 ± 0.55 vs 3.35 ± 0.65, p > 0.05 **OA knees**  UPS pre-walk > supine  5.02 ± 1.67 > 3.96 ± 1.3, p < 0.001 | N/A | **Supine**  OA knee ~ healthy  3.96 ± 1.3 vs 3.35 ± 0.65, p = 0.802 **Standing pre-walk** OA knee > healthy  5.02 ± 1.67 vs 3.58 ± 0.55, p = 0.014 |
| Yanagisawa 2014 [53] | **Healthy knee** Standing ~ supine 2.95 ± 0.93 vs 2.40 ± 0.87, p > 0.05 **OA knee** Standing ~ supine  7.00 ± 2.65 vs 6.12 ± 2.57, p > 0.05 | N/A | **Supine**  OA knee > healthy 6.12 ± 2.57 vs 2.40 ± 0.87, p < 0.001 **Standing**  OA knee > healthy 7.00 ± 2.65 vs 2.95 ± 0.93, p < 0.001 |
| Yanagisawa 2015 [52] | **Healthy knee** Standing ~ supine  2.8 ± 1.1 vs 2.1 ± 1.0, p > 0.05 **OA knee** Standing ~ supine  6.3 ± 7.7 vs 5.0 ± 2.4, p > 0.05 | N/A | **Supine**  OA knee > healthy 5.0 ± 2.4 vs 2.1 ± 1.0, p < 0.01 **Standing**  OA knee > healthy 6.3 ± 7.7 vs 2.8 ± 1.1 p < 0.01 |
| Murakami 2017 [41] | **Pre OA: Healthy knee** Standing > Supine 4.0 ± 1.1 vs 3.6 ± 1.0, p < 0.0001 **Early OA: K/L Grade 2** Standing > Supine  6.2 ± 1.1 vs 5.6 ± 1.0, p < 0.001 | N/A | **Supine**  K/L Grade 2 > Healthy knee 5.6 ± 1.0 vs 3.6 ± 1.0, p < 0.05 **Standing**  K/L Grade 2 > Healthy knee 6.2 ± 1.1 vs 4.0 ± 1.1, p < 0.05 |
| Ishii 2017 [36] | **Healthy knee** Standing > Supine  1.48 ± 0.72 vs 1.31 ± 0.73, p < 0.05 **OA knee** Standing > Supine 6.12 ± 1.81 vs 5.45 ± 1.77, p < 0.05 | N/A | **Supine** OA knee > Healthy 5.45 ± 1.77 vs 1.31 ± 0.73, p < 0.05 **Standing**  OA knee > Healthy 6.12 ± 1.81 vs 1.48 ± 0.72, p < 0.05 |
| Achtnich 2018 [29] | **Healthy knees** Loaded > Unloaded 1.9 ± 0.9 vs. 1.1 ± 0.5, p < 0.05 | N/A | N/A |
| Diermeier 2019 [32] | **Final stage of marathon (270.5km)**  Standing (FWB) ~ Supine (NWB)  3.1 ± 0.6 vs 2.7 ± 0.7, p > 0.05 | N/A | N/A |
| Karpinski 2019 [40] | **Healthy knees** Standing > Supine 2.3 ± 0.4 vs 1.3 ± 0.3, p < 0.001 **Meniscal root tear** Standing ~ Supine  3.7 ± 0.9 vs 3.6 ± 1.0, p > 0.5 | **Supine**  Meniscal root tear > healthy  3.6 ± 1.0 vs 1.3 ± 0.3, p < 0.05 **Standing**  Meniscal root tear > healthy 3.7 ± 0.9 vs 2.3 ± 0.4, p < 0.05 | N/A |
| Ozdemir 2019 [44] | **Healthy knee**  Standing ~ Supine  3.51 ± 0.71 vs 3.13 ± 0.6 **K/L 2**: Standing ~ Supine  6.37 ± 1.06 vs 5.45 ± 0.93 **K/L 3**: Standing ~ Supine  8.88 ± 1.48 vs 7.25 ± 1.19 **K/L 4**: Standing ~ Supine  10.4 ± 1.44 vs 8.6 ± 1.24 | N/A | **Supine** K/L 1 ~ Healthy: 3.72 ± 0.71 vs 3.13 ± 0.6 K/L 2 > Healthy: 5.45 ± 0.93 vs 3.13 ± 0.6, p < 0.05 K/L 3 > Healthy: 7.25 ± 1.19 vs 3.13 ± 0.6, p < 0.05 K/L 4 > Healthy: 8.6 ± 1.24 vs 3.13 ± 0.6, p < 0.05 **Standing**  K/L 1 ~ Healthy: 4.31 ± 0.75 vs 3.51 ± 0.71  K/L 2 > Healthy: 6.37 ± 1.06 vs 3.51 ± 0.71, p < 0.05 K/L 3 > Healthy: 8.88 ± 1.48 vs 3.51 ± 0.71, p < 0.05 K/L 4 > Healthy: 10.4 ± 1.44 vs 3.51 ± 0.71,p < 0.05 |
| Elkwesny 2020 [33] | **Primary OA** Standing > Supine 6 ± 1.21 vs 4.31 ± 1.52, p < 0.05 | N/A | N/A |
| Ishii 2020 [37] | **KL 2:** Standing > Supine  3.60 ± 1.48 vs 2.67 ± 1.44, p < 0.05 **KL 3/4**: Standing > Supine  6.32 ± 1.71 vs 5.65 ± 1.72, p < 0.05 | N/A | N/A |
| Ishii 2020* [39] | N/A | N/A | **Standing** OA > healthy 1.5 ± 0.3 vs. 0.8 ± 0.1, p < 0.05 |
| Shimozaki 2020 [50] | **Healthy knees** Standing > Supine  1.7 ± 0.6 vs 0.9 ± 0.6, p < 0.05 | N/A | N/A |
| Cho JC 2021[30] | **Healthy knees** Standing > Supine 2.8 ± 0.8 vs 2.3 ± 0.5, p < 0.05 | N/A | N/A |
| Reisner 2021 [48] | **Healthy knees** Standing ~ supine  3.4 ± 0.7 vs 3.0 ± 0.7, p > 0.05 **OA knee** Standing ~ supine  4.5 ± 1.6 vs 3.9 ± 1.7, p > 0.05 | N/A | **Supine**   OA > healthy  3.9 ± 1.7 vs 3.0 ± 0.7, p < 0.05 **Standing**  OA > healthy 4.5 ± 1.6 vs 3.4 ± 0.7, p < 0.05 |
| Winkler 2021 [51] | **Healthy knees** Supine > Standing  2.6 ± 0.5 vs 2.2 ± 0.5, p < 0.001 **ACLR and LM repair** Standing ~ Supine  2.9 ± 1.0 vs 2.8 ± 0.5, p = 0.728 | N/A | N/A |
| Zeitoun 2021[55] | **Healthy knees** Standing > Supine  2.71 ± 0.43 vs 1.95 ± 0.49, p = 0.001 **Meniscal tear** Standing > Supine 3.82 ± 0.84 vs 2.50 ± 0.65, p = 0.001 | **Supine**  Meniscal tear ~ healthy  2.50 ± 0.65 vs 1.95 ± 0.49 **Standing**  Meniscal tear > healthy 3.82 ± 0.84 vs 2.71 ± 0.43, p < 0.05 | N/A |
| Falkowski 2022 [34] | **Healthy knee** Standing > Supine 1.59 ± 1.07 vs 0.77 ± 0.87,  p = 0.001 **Meniscal degeneration** Standing > Supine 2.30 ± 0.90 vs 1.65 ± 1.23, p = 0.001 **Meniscal tear** Standing > supine 2.31 ± 1.21 vs 1.63 ±. 1.19, p = 0.001 | **Supine** MD > Healthy 1.65 ± 1.23 vs 0.77 ± 0.87, p < 0.05 MT > Healthy  1.63 ±. 1.19 vs 0.77 ± 0.87, p < 0.05 **Standing**  MD > Healthy 2.30 ± 0.90 vs 1.59 ± 1.07, p < 0.05 MT > Healthy 2.31 ± 1.21 vs 1.59 ± 1.07, p < 0.05 | N/A |
| Ishii 2023 [38] | **Primary OA** Dynamic walking > Supine 5.6 ± 2.4 vs 4.9 ± 2.2, p < 0.05 | N/A | N/A |

**Additional file 3.** The use of US in detecting changes in extrusion between both loading and unloading positions and different knee states. MD, Meniscal Degeneration

|  |  |
| --- | --- |
| Study | Change in MME (mm) with US between different mild and moderate to severe OA |
| Ko 2007 [8] | **Standing**  **KL 3/4 > KL 1/2**: 5.6 ± 1.8 vs 2.9 ± 0.7, p < 0.001 |
| Kawaguchi 2012 [19] | **Supine: K3 > KL1:** 6.83 ± 1.21 vs 3.84 ± 0.9, p < 0.05, **KL 3 > KL 2**: 6.83 ± 1.21 vs 5.18 ± 1.22, p < 0.05  **KL 4 > KL 1**: 8.36 ± 0.87 vs 3.84 ± 0.9, p < 0.05, **KL 4 > KL 2**: 8.36 ± 0.87 vs 5.18 ± 1.22, p < 0.05 **Standing: KL 3 > KL 1**: 7.76 ± 1.17 vs 4.44 ± 1.10, p < 0.05, **KL 3 > KL 2**: 7.76 ± 1.17 vs 5.71 ± 1.39, p < 0.05,  **KL 4 > KL 1**: 9.24 ± 0.88 vs 4.44 ± 1.10, p < 0.05, **KL 4 > KL 2:** 9.24 ± 0.88 vs 5.71 ± 1.39, p < 0.05 |
| Yanagisawa 2014 [53] | **Supine: KL 3 > KL 1**: 6.31 ± 1.94 vs 2.66 ± 1.03, p < 0.001, **KL 3 > KL 2**: 6.31 ± 1.94 vs 3.58 ± 1.35, p < 0.001  , **KL 4 > KL 1**: 8.21 ± 2.1 vs 2.66 ± 1.03, p < 0.001, **KL 4 > KL 2**: 8.21 ± 2.1 vs 3.58 ± 1.35, p < 0.001 **Standing : KL 3 > KL 1**: 7.33 ± 1.9 vs 3.26 ± 1.1, **KL 3 > KL 2**: 7.33 ± 1.9 vs 4.27 ± 1.46, p < 0.001 **KL 4 > KL 1**: 9.13 ± 2.1 vs 3.26 ± 1.1, p < 0.001, **KL 4 > KL 1:** 9.13 ± 2.1 vs 4.27 ± 1.46, p < 0.001 |
| Ozdemir 2019 [44] | **Supine: KL 3 > KL 1**: 7.25 ± 1.19 vs 3.72 ± 0.71, p < 0.05, **KL 3 > 2:** 7.25 ± 1.19 vs 5.45 ± 0.93, p < 0.05 **KL 4 > 1:** 8.6 ± 1.24 vs 3.72 ± 0.71, p < 0.05, **KL 4 > 2:** 8.6 ± 1.24 vs 5.45 ± 0.93, p < 0.05 **Standing: KL 3 > 1**: 8.88 ± 1.48 vs 4.31 ± 0.75, p < 0.05, **KL 3 > KL 2**: 8.88 ± 1.48 vs 6.37 ± 1.06, p < 0.05 **KL 4 > 1**: 10.4 ± 1.44 vs 4.31± 0.75, p < 0.05, **KL 4 > KL 2:** 10.4 ± 1.44 vs 6.37 ± 1.06, p < 0.05 |
| Ishii 2020 [37] | **Supine: KL 3/4 > KL 2**: 5.65 ± 1.72 vs 2.67 ± 1.44, p < 0.05 **Standing: KL 3/4 > KL 2**: 6.32 ± 1.71 vs 3.60 ± 1.48, p < 0.05 |

**Additional file 4** The use of US in detecting changes in extrusion between mild (K/L 1 or 2) and moderate-severe (K/L 3 or 4) OA knees.
